# Hepatobiliary phenotype of individuals with chronic intestinal disorders

**DOI:** 10.1038/s41598-021-98843-7

**Published:** 2021-10-07

**Authors:** Jessica Voss, Carolin V. Schneider, Moritz Kleinjans, Tony Bruns, Christian Trautwein, Pavel Strnad

**Affiliations:** Medical Clinic III, Gastroenterology, Metabolic Diseases and Intensive Care, University Hospital RWTH Aachen, Pauwelsstr. 30, 52074 Aachen, Germany

**Keywords:** Hepatology, Liver diseases, Liver cancer, Liver cirrhosis, Non-alcoholic fatty liver disease, Primary sclerosing cholangitis, Inflammatory bowel disease, Gastroenterology, Coeliac disease, Inflammatory bowel disease

## Abstract

Despite the known functional relationship between the gut and the liver, the clinical consequences of this circuit remain unclear. We assessed the hepatobiliary phenotype of cohorts with celiac disease (CeD), Crohn´s disease (CD) and ulcerative colitis (UC). Baseline liver function tests and the frequency of hepatobiliary diseases were analyzed in 2377 CeD, 1738 CD, 3684 UC subjects and 488,941 controls from the population-based UK Biobank cohort. In this cohort study associations were adjusted for age, sex, BMI, diabetes, and alcohol consumption. Compared to controls, cohorts with CeD, but not CD/UC displayed higher AST/ALT values. Subjects with CD/UC but not CeD had increased GGT levels. Elevated ALP and cholelithiasis were significantly more common in all intestinal disorders. Non-alcoholic steatohepatitis and hepatocellular carcinoma (HCC) were enriched in CeD and CD (NASH: ^t^aOR = 4.9 [2.2–11.0] in CeD, aOR = 4.2 [1.7–10.3] in CD, HCC: aOR = 4.8 [1.8–13.0] in CeD, aOR = 5.9 [2.2–16.1] in CD), while cholangitis was more common in the CD/UC cohorts (aOR = 11.7 [9.1–15.0] in UC, aOR = 3.5 [1.8–6.8] in CD). Chronic hepatitis, autoimmune hepatitis (AIH) and cirrhosis were more prevalent in all intestinal disorders. In UC/CD, a history of intestinal surgery was associated with elevated liver enzymes and increased occurrence of gallstones (UC: aOR = 2.9 [2.1–4.1], CD: 1.7 [1.2–2.3]). Our data demonstrate that different intestinal disorders predispose to distinct hepatobiliary phenotypes. An increased occurrence of liver cirrhosis, NASH, AIH and HCC and the impact of surgery warrant further exploration.

## Introduction

The term “gut-liver axis” reflects the close bidirectional relationship between gut including microbiota and the liver. This crosstalk is achieved by an exchange of factors that is enabled by a joint vascular system as well as by hepatobiliary circulation of the bile and its constituents^1^. In particular, bile and bile acids are produced in the liver, secreted into the duodenum and taken up in the terminal ileum returning to the liver. Bile acids are modified by microbiota, but also have an impact on microbiome composition^2^. While multiple experimental data demonstrate the importance of the gut-liver axis, its impact in a clinical setting is still only partially understood^3–5^.

Among the prevalent intestinal disorders, celiac disease (CeD) constitutes an immune-based small intestinal disorder developing in response to dietary gluten^6,7^. Untreated CeD often goes along with elevated transaminases and in some cases with mild hepatic inflammation known as celiac hepatitis, which is associated with the presence of a leaky gut, that leads to translocation of microbial products into the portal blood system^6,8^. Notably, liver enzymes in CeD correlate with the extent of intestinal damage and mostly normalize when the patients adhere to gluten-free diet^9,10^. Several studies suggested that CeD is associated with an increased risk of cirrhosis, however, the magnitude of this risk remains controversial^11,12^. Additionally, CeD is associated with various autoimmune disorders, including autoimmune hepatitis (AIH)^13,14^.

Ulcerative colitis (UC) and Crohn´s disease (CD) are multifactorial, chronic inflammatory disorders, known as inflammatory bowel diseases (IBD), characterized by an impaired epithelial barrier and deregulated immune responses. UC affects the colon, whereas CD can damage the entire gastrointestinal tract, but is often particularly severe in the terminal ileum^15,16^. Accordingly, a colectomy constitutes a curative treatment option in therapy-refractory UC, while intestinal resections in CD are typically performed due to complications such as strictures, fistulas or abscesses^15,16^. UC and to smaller extent CD go along with primary sclerosing cholangitis (PSC), a progressive destruction of the biliary tree. Several intestinal factors, such as intestinal microbiome and its byproducts, have been implicated in the pathogenesis of PSC^5,17,18^.

Loss of biliary products, which is precipitated by inflammation and/or removal of the terminal ileum, promotes the formation of gallstones that is particularly common in CD individuals^19–21^. Terminal ileum also produces a variety of important metabolic factors such as GLP-1 and therefore, CD was suggested to increase the susceptibility to liver steatosis^22,23^. Development of liver steatosis in CD/UC is further supported by intake of corticosteroids that are utilized to induce remission in acutely sick individuals^24,25^. Despite these observations, the risk to develop terminal liver disease, including liver cirrhosis and/or hepatocellular carcinoma (HCC) remains unclear^26–28^.

Collectively, the liver injury in CeD vs. CD/UC is thought to arise due to distinct mechanisms, i.e. gluten-related immune reaction plus increased leakiness of upper gastrointestinal tract in CeD vs. involvement of terminal ileum/colon with a much higher bacterial density, role in bile acid metabolism plus drug-related injury in CD/UC. While a significant amount of research has been performed on chronic intestinal disorders and the associated hepatobiliary phenotypes, much of the data come from tertiary centers and the exact liver phenotypes seen in general population remain unclear. Moreover, the existing studies were unable to side-by-side compare different intestinal disorders and thereby to dissect their specific effects^8,11,22,28^. Therefore we used the UK biobank, a large community-based sample of ~ 500,000 individuals from the United Kingdom with deep genetic, physical, and health data, to better unravel the impact of these diseases on the gut-liver axis^29^.

## Methods

### Population-based UK biobank participants

The ‘UK biobank’ (UKB) is a population-based cohort study built up in the United Kingdom from 2006 to 2010. In this period approximately 500,000 individuals from across the United Kingdom, aged 37 to 73 years at baseline, were recruited and registered with the UK National Health Service. At baseline visit, all participants gave informed consent for genotyping and data linkage to medical reports. They provided socio-demographic and clinical data, blood samples and physiological measures in an initial examination, which was the basis for our study. ICD-10 codes (international classification of diseases, 10th revision) were obtained from medical records from the year 1996 on to identify diagnoses. Participants with chronic hepatitis B or C, pathological alcohol consumption (> 60 g/d in men or > 40 g/d in women) or co-existence of IBD/CeD (n = 578) were excluded (in total n = 5771, Supplementary Fig. 1).

2377 individuals with celiac disease (CeD), 3684 with ulcerative colitis (UC) and 1738 with Crohn’s disease (CD) were included in our study. We compared liver enzymes in the blood as well as liver-related ICD-codes between cohorts with CeD, UC, CD and controls. For each disease entity, we compared cohorts with and without cirrhosis to assess the underlying risk factors. Bowel resection was defined as operation codes 1464, 1459, 1461, 1462, and 1465. The presence of the following primary ICD10 codes was evaluated: Celiac disease (K90.0), ulcerative colitis (K51.0–9), Crohn’s disease (K50.0–9), cirrhosis (K74.6), non-alcoholic steatohepatitis (NASH)(K75.8), chronic hepatitis (K73), primary liver cancer (C22.0), cholelithiasis, cholecystitis (K80, K81) and AIH (K75.4). The study has been approved by the UKB Access Committee (Project #47527). The manuscript is based solely on the analysis of pseudonymized data obtained from the UK Biobank Resource under Application Number 47527. The authors were not in contact with the described individuals nor had they access to their personal data. The data were reported as described by the STROBE guidelines.

### Statistical analysis

Kolmogorov–Smirnov-test was used to assess normal distribution. All continuous variables were analyzed by unpaired, two-tailed t-tests or Mann–Whitney U test, and by a multivariable model to account for relevant confounders. As a result, all these variables were shown as mean ± standard deviation (normal distribution) or median [IQR] (non-normal distribution). All categorical variables were displayed as relative (%) frequencies and the corresponding contingency tables were analyzed using the Chi-square test.

All analyses were adjusted for age, sex, BMI, presence of diabetes mellitus, and mean alcohol consumption via multivariable logistic or linear regression. Odds ratios (ORs) were presented with their corresponding 95% confidence intervals (CI) given in brackets. Multivariable logistic regression was performed to test for independent associations. Differences were statistically significant when p < 0.05. The data were analyzed using SPSS Statistics version 26 (IBM; Armonk, NY, USA). Data presentation was performed using Prism version 8 (GraphPad, LaJolla, CA, USA).

**Table 1:** Liver phenotype in UK Biobank cohorts with celiac disease, Crohn’s disease or ulcerative colitis compared to controls.

	Controls *n* = *488.941*	Celiac disease (CeD) *n* = *2 377*	Crohn (CD) *n* = *1 738*	Ulcerative colitis (UC) *n* = *3 684*	p-value CeD vs. Controls	p-value UC vs. Controls	p-value CD vs. Controls
**Characteristics**					Univariate	Univariate	Univariate
Age (years)	56.5 ± 8.1	58.0 ± 7.8	56.6 ± 8.1	57.6 ± 7.9	**< 0.001**	**< 0.001**	0.61
Women (%)	54	65	57	48	**< 0.001**	**< 0.001**	*0.020*
BMI (kg/m^2^)	27.4 ± 4.8	26.1 ± 4.8	27.0 ± 4.9	27.6 ± 4.7	**< 0.001**	0.13	**< 0.001**
Alcohol (g/d)	8.8 ± 10.1	6.6 ± 8.5	6.7 ± 9.1	8.4 ± 10.1	**< 0.001**	**0.031**	**< 0.001**
Diabetes mellitus (%)	5.3	5.2	6.9	7.5	0.81	**< 0.001**	*0.002*
Smoking current	10.4	8.0	17.6	7.9	**< 0.001**	** 0.001**	**< 0.001**
Previous	34.3	33.7	39.5	45.8			
Never	54.9	57.9	42.6	45.9			
**Liver-related ICD10codes**					Multivariable	Multivariable	Multivariable
Cirrhosis (%)	0.21	0.55	0.81	0.84	**< 0.001**^**a**^	**< 0.001**^**b**^	**< 0.001**^**c**^
Chronic hepatitis (%)	0.03	0.29	0.23	0.16	**< 0.001**^**d**^	**< 0.001**^**e**^	**< 0.001**^**f**^
AIH (%)	0.04	0.25	0.35	0.24	**< 0.001**^**g**^	**< 0.001**^**h**^	**< 0.001**^**i**^
NASH (%)	0.07	0.25	0.29	0.16	**< 0.001**^**j**^	0.065	*0.002*^*k*^
Cholelithiasis (%)	3.9	6.1	10.8	5.9	**< 0.001**^**l**^	**< 0.001**^**m**^	**< 0.001**^**n**^
Cholecystitis (%)	0.7	1.1	2.0	1.2	0.071	*0.007*^*o*^	**< 0.001**^**p**^
Cholangitis (%)	0.14	0.29	0.52	1.85	0.061	**< 0.001**^**q**^	**< 0.001**^**r**^
HCC (%)	0.04	0.17	0.23	0.08	*0.002*^*s*^	0.34	**< 0.001**^**t**^
CCA (%)	0.04	0.04	0.17	0.11	0.78	0.21	*0.042*^*u*^
**Surgery**							
Bowel resection (%)	0.9	1.2	31.5	10.4	0.36	**< 0.001**^**v**^	**< 0.001**^**w**^
Small bowel resection (%)	0.1	0.3	11.2	1.0	0.10	**< 0.001**^**x**^	**< 0.001**^**y**^

Quantitative measures are expressed as mean with standard deviation or relative frequency (%). Multivariable analyses were adjusted for age, sex, BMI, presence of diabetes mellitus, and mean alcohol consumption. p-values 0.001–0.05 in italics and p-value < 0.001 in bold. *BMI* body mass index, *CCA* cholangiocarcinoma, *NASH* Non-alcoholic steatohepatitis. ^a^aOR = 3.59[2.11–6.10]; ^b^aOR = 3.67[2.55–5.26]; ^c^aOR = 3.82[2.20–6.65]; ^d^aOR = 8.51[3.97–18.22]; ^e^aOR = 4.80[2.10–10.86]; ^f^aOR = 6.51[2.41–17.59]; ^g^aOR = 5.50[2.43–12.43]; ^h^aOR = 6.08[3.11–11.88]; ^i^aOR = 7.87[3.48–17.76]; ^j^aOR = 4.87[2.16–11.00]; ^k^aOR = 4.22[1.74–10.27]; ^l^aOR = 1.66[1.40–2.00]; ^m^aOR = 1.57[1.37–1.81]; ^n^aOR = 3.00[2.57–3.52]; ^o^aOR = 1.52[1.12–2.07]; ^p^aOR = 2.70[1.92–3.80]; ^q^aOR = 11.73[9.11–15.01]; ^r^aOR = 3.52[1.82–6.81]; ^s^aOR = 4.79[1.77–12.96]; ^t^aOR = 5.93[2.20–16.05]; ^u^aOR = 3.26[1.04–10.19]; ^v^aOR = 12.632[11.02–13.77]; ^w^aOR = 53.67[48.11–59.87]; ^x^aOR = 7.90[5.63–11.09]; ^y^aOR = 102.75[86.45–122.1].

## Results

### Characterization of study cohort

Among 497,404 participants in the UK biobank, we identified 2377 individuals with CeD, 1738 with CD and 3684 with UC (Supplementary Fig. 1). The CeD or UC cohorts were slightly older than controls and the CD cohort. 65% of individuals with CeD and 48% of the UC cohort were female compared to 54% of controls (Table 1). Participants from all disease subgroups reported lower average alcohol consumption than controls. Individuals with CeD and CD had a lower average BMI than controls (Table 1).

### Serum liver enzyme concentrations in chronic intestinal disorders

**Figure 1:**
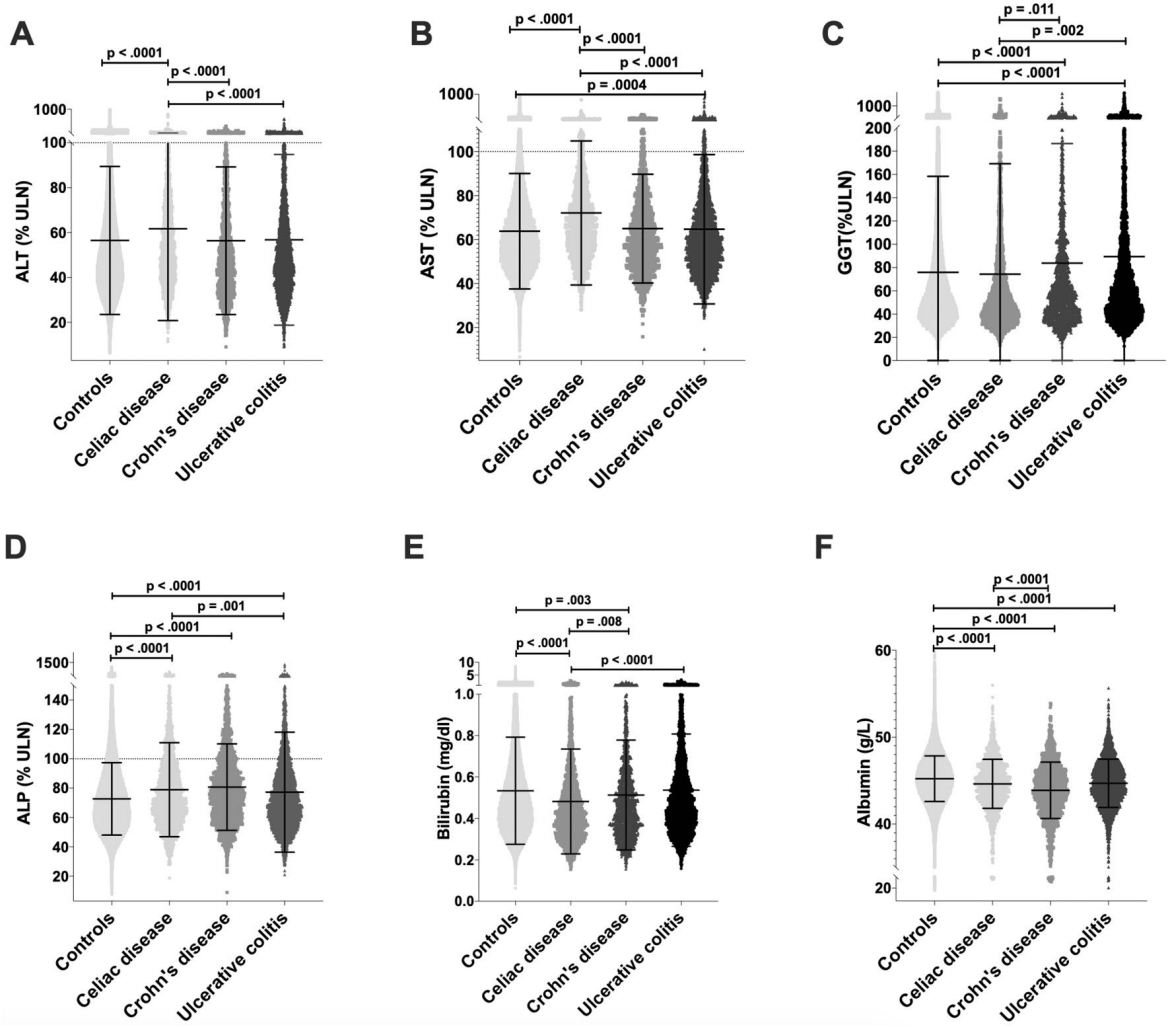
Liver related parameters in individuals with celiac disease, Crohn’s disease, and ulcerative colitis compared to healthy controls. 488,941 control participants. 2377 subjects with diagnosis of celiac disease. 1738 individuals with Crohn’s disease and 3684 participants with ulcerative colitis underwent laboratory analysis. p values were adjusted for age, sex, BMI, alcohol consumption and diabetes mellitus using a linear regression model. Scatter plots of serum level of aspartate aminotransferase (AST; **A**), alanine aminotransferase (ALT; **B**), gamma glutamyl transferase (GGT; **C**); and alkaline phosphatase (ALP; **D**) are shown, all normalized to the sex-specific upper limit of normal (ULN) (marked as dotted line). Scatter plot of serum levels of bilirubin (**E**) and albumin (**F**).

**Figure 2:**
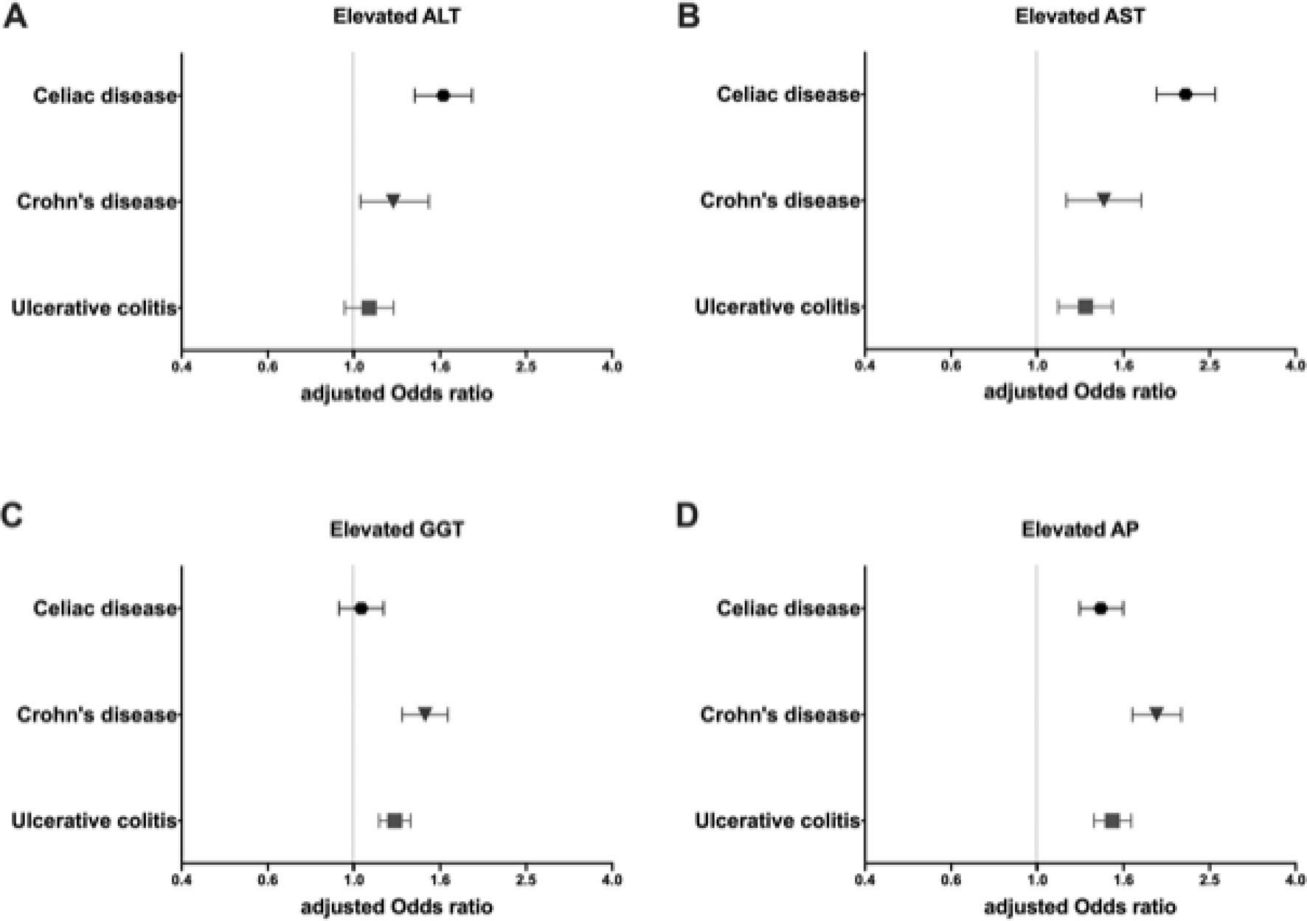
Odds ratios to present with elevated serum ALT, AST, GGT or ALP levels in UK Biobank cohorts with celiac disease, Crohn’s disease, and ulcerative colitis compared to controls. Adjusted odds ratios (OR) with their corresponding 95% confidence intervals (CI) are shown for alanine aminotransferase (ALT; **A**), aspartate aminotransferase (AST; **B**), gamma glutamyl transferase (GGT; **C**) and alkaline phosphatase (ALP; **D**). The risk to display levels higher than the corresponding sex-dependent upper limit of normal (ULN) was compared to the respective controls. Odds ratios were adjusted for age, sex, BMI, alcohol consumption and diabetes mellitus.

Mean alanine aminotransferase (ALT) and aspartate aminotransferase (AST) concentrations in the CeD cohort were significantly higher than in controls (Supplementary Table 1, Fig. 1A,B). Accordingly, CeD participants more frequently displayed elevated AST/ALT values than controls (ALT: aOR = 1.62[1.39–1.89]; p < 0.001; AST: aOR = 2.24[1.94–2.58]; p < 0.001) (Supplementary Table 1; Fig. 2). While the CD or UC cohorts were also more likely to have AST elevations above the upper limit of normal than controls (Supplementary Table 1), the corresponding odds ratios were substantially lower (Fig. 2). In contrast, participants with IBD showed significantly higher gamma-glutamyl transferase (GGT) concentrations and more often had elevated GGT than controls (UC: aOR = 1.25[1.15–1.36]; p < 0.001; CD: aOR = 1.47[1.30–1.66], p < 0.001; Supplementary Table 1; Figs. 1C, 2C). Notably, all cohorts with intestinal disorders including CeD, CD, and UC displayed significantly higher alkaline phosphatase (ALP) concentrations than controls (Supplementary Table 1; Fig. 1D) and more often had increased ALP concentrations (CeD: aOR = 1.41[1.26–1.59]; UC: aOR = 1.50 [1.36–1.66]; CD: aOR = 1.90 [1.67–2.17]; all p < 0.001, Supplementary Table 1, Fig. 2D). In the vast majority, only mild elevations of AST/ALT/ALP were seen (i.e. ≤ 2 × ULN), whereas moderately elevated GGT levels (i.e. ≥ 2 × ULN) were detected in 4–6% of all individuals and were more common in the UC/CD cohorts (Supplementary Table 2). While the number of patients with elevated total serum bilirubin was comparable in all groups, serum albumin concentrations were significantly lower in all disease cohorts with lowest concentrations seen in the CD cohort. (Supplementary Table 1, Fig. 1E,F).

### Liver enzyme serum concentrations in selected subgroups

**Figure 3:**
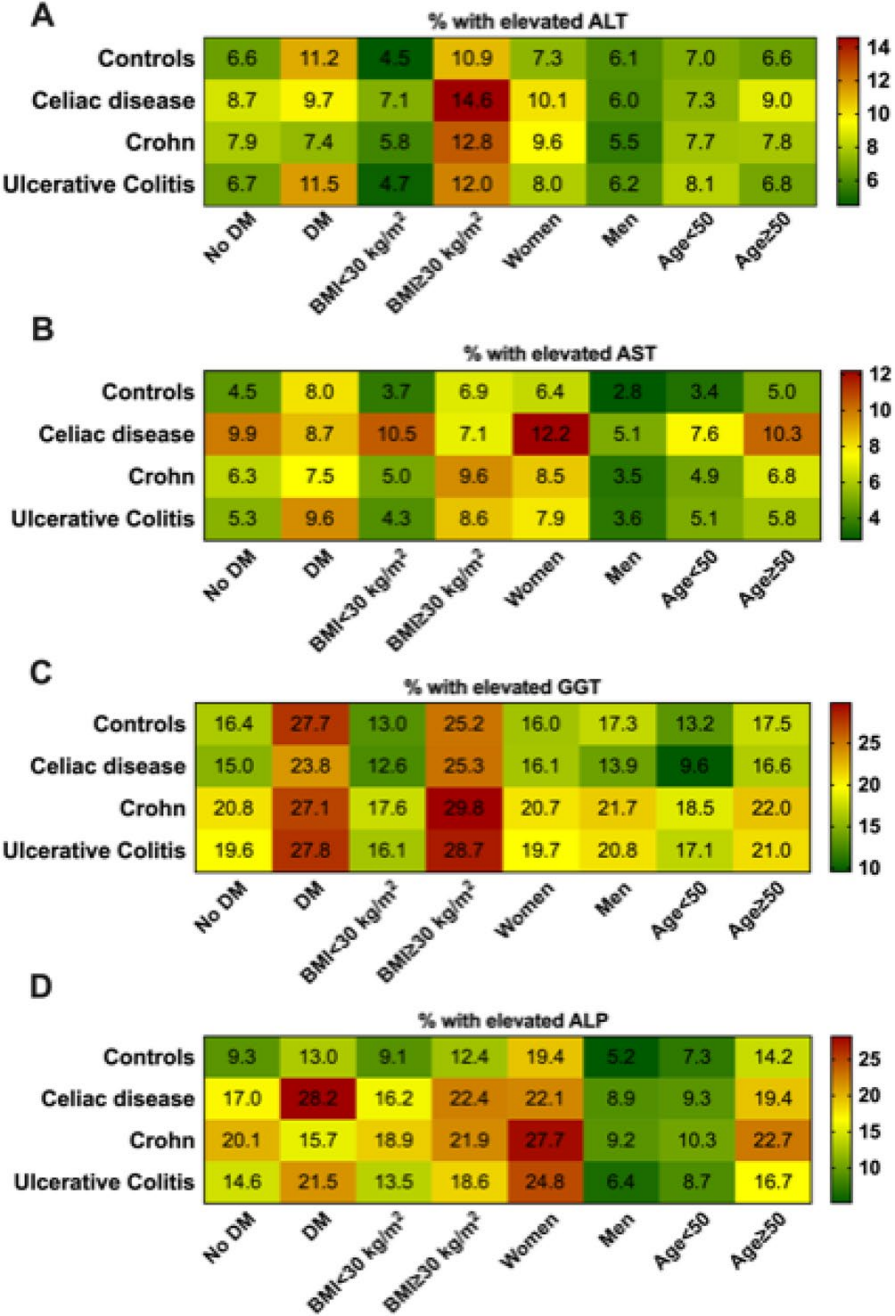
Frequency of elevated liver enzymes in subcohorts of patients with celiac disease, Crohn’s disease, and ulcerative colitis compared to healthy controls. Relative frequencies (%) are shown and visualized by a color coding (right panel). *ALT* alanine aminotransferase, *AST* aspartate aminotransferase, *ALP* alkaline phosphatase, *AST* aspartate aminotransferase, *GGT* gamma-glutamyl transferase, *BMI* body mass index (kg/m^2^), *DM* diabetes mellitus.

Next, we analyzed factors associated with elevated liver enzymes. In all investigated intestinal diseases, females more often displayed elevated transaminase serum concentrations than males. Individuals with IBD who had a BMI > 30 kg/m^2^ more frequently demonstrated elevated AST/ALT levels than their non-obese counterparts. Somewhat surprisingly, diabetes was associated with elevated AST/ALT values in the UC cohort, but not in the CD or CeD cohorts (Fig. 3). Individuals who had diabetes, were obese or of higher age, and more frequently displayed elevated GGT serum concentrations irrespective of the underlying intestinal disease (Fig. 3). Females and participants aged 50 years or older often presented with elevated ALP concentrations, whereas the impact of diabetes and obesity was less evident (Fig. 3). Notably, the observed changes reflected mostly the alterations seen in the control group (Fig. 3).

### Liver-related diagnoses in cohorts with chronic intestinal disorders

**Figure 4:**
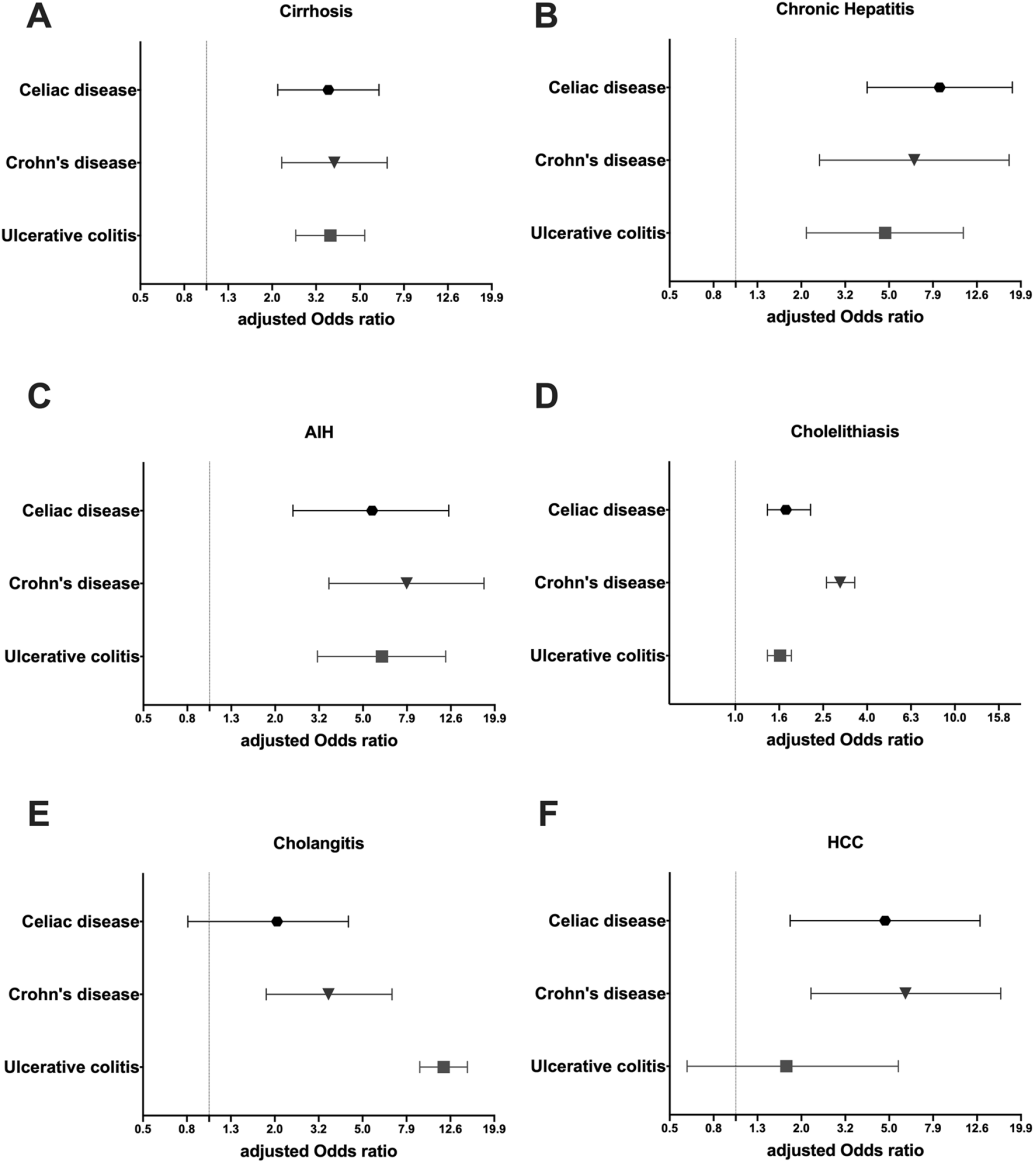
Odds ratios to display selected ICD10 diagnoses in cohorts with celiac disease, Crohn’s disease, and ulcerative colitis compared to controls. (**A**) Cirrhosis, (**B**) chronic hepatitis (K73), (**C**) autoimmune hepatitis (AIH), (**D**) cholelithiasis, (**E**) cholangitis, (**F**) hepatocellular carcinoma (HCC). Adjusted odds ratios (OR) with their corresponding 95% confidence intervals (CI) are shown. Odds ratios were adjusted for age, sex, BMI, alcohol consumption and diabetes mellitus.

To determine, whether the differences in liver-related parameters translate into clinically relevant diseases, we analyzed the occurrence of the most relevant hepatobiliary ICD codes in the described cohorts (Table 1). Among the hepatic disorders, individuals from all disease subgroups more frequently displayed cirrhosis, chronic hepatitis or autoimmune hepatitis (Table 1, Fig. 4). While the odds ratios for developing cirrhosis ranged between 3 and 4 compared to controls, even higher odds were seen for chronic hepatitis (ORs between 4 and 9) and autoimmune hepatitis (ORs between 5 and 8).

Among the biliary diseases investigated, cholelithiasis was more common in all disease subgroups compared to controls and was particularly frequent in the CD cohort (Table 1, Fig. 4D). As another evidence highlighting the importance of small bowel in the pathogenesis of gallstone formation, cholelithiasis was nearly twice as common in a CD subcohort with isolated small bowel affection compared to the subcohort with isolated affection of the colon (Supplementary Table 3). Notably, none of the other assessed parameters differ between the subcohorts (Supplementary Table 3). Cholecystitis was much less common, but displayed a similar distribution pattern. In line with the published data, the ICD code cholangitis (that includes primary sclerosing cholangitis) was most common in UCs, but also significantly overrepresented in CDs when compared to controls (Table 1, Fig. 4E). Likely due to its dismal prognosis, the occurrence of cholangiocarcinoma (CCA) in UK participants was very low and when compared between the subgroups, the ICD code was significantly elevated only in the CD cohort, (CD: OR = 3.26[1.04–10.19]; p = 0.042, Table 1). In contrast, the diagnosis of HCC was significantly more common in the CD and CeD cohort, but not in UC participants (CeD: aOR = 4.79[1.77–12.96]; CD: aOR = 5.93[2.20–16.05]; both p < 0.01) (Fig. 4F, Table 1).

**Table 2:** Comparison of UK Biobank cohorts with ulcerative colitis that did/did not undergo bowel resection.

	Ulcerative colitis *without bowel* *resection* *n* = *3300*	Ulcerative colitis *with bowel resection* *n* = *384*	p-value (univariate analysis)	p-value (multivariable analysis)
**Characteristics**				
Age (years)	57.5 ± 8.0	58.2 ± 7.3	0.085	
Women (%)	48	45	0.28	
BMI (kg/m^2^)	27.6 ± 4.6	27.0 ± 4.7	**0.012**	
Alcohol (g/d)	8.6 ± 10.3	7.3 ± 9.0	**0.011**	
Diabetes mellitus (%)	7	10	0.054	
**Liver status**				
ALT (% of ULN)	55.2 ± 34.2	65.5 ± 43.7	**< 0.001**	**< 0.001**
ALT ≥ ULN (%)^a^	6.6	11.0	**0.003**	**0.002**
AST (% of ULN)	63.2 ± 26.1	74.1 ± 59.5	**0.001**	**< 0.001**
AST ≥ ULN (%)^b^	5.0	11.6	**< 0.001**	**< 0.001**
GGT (% of ULN)	83.5 ± 115.2	134.1 ± 189.2	**< 0.001**	**< 0.001**
GGT ≥ ULN (%)^c^	18.6	35.1	**< 0.001**	**< 0.001**
ALP (% of ULN)	75.9 ± 35.0	105.3 ± 94.4	**0.001**	**< 0.001**
ALP ≥ ULN (%)^d^	14.3	22.3	**0.001**	**< 0.001**
**ICD10 codes**				
Cholelithiasis^e^	5.12	13.02	**< 0.001**	**< 0.001**
Cholangitis^f^	1.09	2.34	**< 0.001**	**< 0.001**

Quantitative measures are expressed as mean with standard deviation or relative frequency (%). All analyses were adjusted for age, sex, BMI, presence of diabetes mellitus, and mean alcohol consumption. p-values <0.05 are highlighted in bold. ^a^unadjusted OR = 1.73[1.21–2.48], adjusted OR = 1.83[1.25–2.67]; ^b^unadjusted OR = 2.49[1.73–3.59], adjusted OR = 2.45[1.69–3.53]; ^c^unadjustedOR = 2.36[1.86–3.00], adjusted OR = 2.64[2.06–3.38]; ^d^OR = 1.71[1.31–2.24], adjusted OR = 1.90[1.43–2.54]; ^e^unadjusted OR = 3.20[1.85–5.54], adjusted OR = 2.89[2.05–4.07]; ^f^unadjusted OR = 2.77[1.98–3.88], adjusted OR = 3.19[1.84–5.52].

**Table 3:** Comparison of UK Biobank cohorts with Crohn’s disease that did/did not undergo bowel resection.

	Crohn *without* *bowel* *resection* *n* = *1191*	Crohn *with* *bowel resection n* = *547*	p-value (univariate analysis)	p-value (multivariable analysis)
**Characteristics**				
Age (years)	56.5 ± 8.2	56.8 ± 7.9	0.55	
Women (%)^a^	55	72	**0.007**	
BMI (kg/m^2^)	27.5 ± 5.1	26.0 ± 4.1	**< 0.001**	
Alcohol (g/d)	7.3 ± 9.4	5.3 ± 7.9	**0.008**	
Diabetes mellitus (%)	7	6	0.17	
**Liver status**				
ALT (% of ULN)	54.1 ± 28.7	60.6 ± 39.7	**0.003**	**< 0.001**
ALT ≥ ULN (%)^b^	6.5	10.6	**0.004**	**< 0.001**
AST (% of ULN)	62.7 ± 22.8	69.1 ± 26.3	**< 0.001**	**< 0.001**
AST ≥ ULN (%)^c^	4.7	9.9	**< 0.001**	**< 0.001**
GGT (% of ULN)	81.5 ± 93.4	96.0 ± 116.9	0.45	0.055
GGT ≥ ULN (%)	20.5	22.5	0.36	0.11
ALP (% of ULN)	79.3 ± 28.5	84.2 ± 31.5	**0.003**	**0.018**
ALP ≥ ULN (%)	18.7	22.1	0.12	0.32
**ICD10 codes**				
Cholelithiasis^d^	9.15	14.26	**0.001**	**0.001**
Cholangitis	0.50	0.55	0.90	0.89

Quantitative measures are expressed as mean with standard deviation or relative frequency (%). All analyses were adjusted for age, sex, BMI, presence of diabetes mellitus, and mean alcohol consumption. p-values <0.05 are highlighted in bold. ^a^Unadjusted OR = 0.75[0.61–0.93]; ^b^unadjusted OR = 1.71[1.18–2.47], adjusted OR = 2.02[1.38–2.98]; ^c^unadjustedOR = 2.22[1.48–3.32], adjusted OR = 2.24[1.50–3.35]; ^d^unadjusted OR = 1.65[1.21–2.25], adjusted OR = 1.70[1.23–2.34].

### The association of bowel resection with liver phenotypes in cohorts with UC and CD

Intestinal resection constitutes an established therapeutic strategy for complications of CD as well as for refractory UC. Therefore, we examined the association between previous intestinal surgery and liver enzyme levels as well as the occurrence of biliary diseases. As expected, bowel resection was less common in the UC than in the CD cohort (32% vs. 10%) and associated with lower BMI and lower reported alcohol consumption (Tables 2 and 3).

Notably, cohorts with CD or UC who had a history of intestinal resection displayed higher AST/ALT values than cohorts without such surgery. Moreover, the UC subcohort also had substantially higher average ALP (105.3% vs. 75.9% of ULN) and GGT concentrations (134.1 vs. 83.5% of ULN), whereas the CD cohort harbored only moderately higher ALP levels (84.2% vs. 79.3% of ULN). In line with the former, part of the UC cohort who underwent bowel resection reported significantly more often cholelithiasis (aOR = 2.89 [2.05–4.07]) and cholangitis (aOR = 3.19 [1.84–5.52]). Individuals with CD who experienced bowel resection more frequently suffered from gallstones (aOR = 1.70 [1.23–2.34] (Tables 2 and 3).

### Subjects with cirrhosis

Since cirrhosis is the major cause of hepatobiliary mortality, we took a closer look at all individuals with this diagnosis. Although the majority of cirrhotics in UK biobank were male, 64% of CD cirrhotics were female (Table 4). Accordingly, females with CD displayed a particularly high odds for HCC compared to female controls (aOR = OR = 7.79 [3.98–15.23]; p < 0.001). Liver enzymes in serum did not significantly differ between cirrhotics with and without the analyzed intestinal disorders (Table 4). The ICD code cholangitis was markedly more common in UC and CD cirrhotics (UC: OR = 37.69 [15.69–90.55]; p < 0.001; CD: OR = 7.37 [1.43–37.94]; p = 0.017), while the diagnosis autoimmune hepatitis was significantly more frequent in UC cirrhotics. Finally, the diagnosis chronic hepatitis was more common in CeD cirrhotics (Table 4).

**Table 4:** Liver phenotype in cirrhotic UK Biobank cohorts with celiac disease, Crohn’s disease or ulcerative colitis compared to controls.

	Controls *with cirrhosis* *n* = *1 012*	Celiac disease (CeD) *with cirrhosis* *n* = *14*	Ulcerative colitis (UC) *with cirrhosis* *n* = *31*	Crohn (CD) *with cirrhosis* *n* = *14*	p-value CeD vs. Controls	p-value UC vs. Controls	p-value CD vs. Controls
**Characteristics**							
Age (years)*	59.1 ± 6.9	59.7 ± 4.8	60.4 ± 6.5	58.1 ± 6.8	0.73	0.31	0.62
Women (%)	31	43	32	64	0.32	0.84	**0.007**^**a**^
BMI (kg/m^2^)	30.3 ± 6.1	28.1 ± 5.1	28.2 ± 4.1	29.4 ± 4.1	0.18	0.059	0.60
Alcohol (g/d)	10.8 ± 14.4	9.7 ± 17.9	8.4 ± 13.9	4.4 ± 8.8	0.77	0.35	0.093
**Risk factors**							
BMI > 30 kg/m^2^	53	50	35	62	0.81	0.051	0.55
Diabetes mellitus (%)	28	21	16	36	0.60	0.15	0.51
**Liver status**							
ALT (% of ULN)	96.4 ± 77.4	122.8 ± 209.0	89.9 ± 96.9	110.8 ± 74.2	0.66	0.66	0.60
ALT ≥ ULN (%)	33.3	15.4	17.2	50.0	0.17	0.069	0.32
AST (% of ULN)	121.4 ± 96.8	160.9 ± 197.4	121.9 ± 136.9	102.7 ± 47.0	0.49	0.98	0.59
AST ≥ ULN (%)	45.8	53.9	31.0	50.0	0.56	0.11	0.81
GGT (% of ULN)	350.7 ± 384.3	346.2 ± 394.4	436.7 ± 506.9	318.2 ± 269.1	0.98	0.24	0.81
GGT ≥ ULN (%)	73.4	75.0	64.0	75.0	0.90	0.30	0.92
ALP (% of ULN)	100.3 ± 70.1	102.3 ± 53.3	153.5 ± 225.5	126.9 ± 83.6	0.90	0.22	0.40
ALP ≥ ULN (%)	33.9	46.2	49.7	50.0	0.35	0.55	0.34
Bilirubin (mg/dl)	0.79 ± 0.59	0.82 ± 0.45	0.93 ± 0.52	0.57 ± 0.26	0.87	0.21	0.30
Albumin (g/l)	43.0 ± 4.2	41.7 ±4.5	42.5 ± 4.5	43.1 ± 1.8	0.35	0.57	0.95
**ICD10**							
Liver-related							
Chronic Hepatitis	3.5	14.3	6.5	7.1	**0.031**^**b**^	0.38	0.46
NASH	12.9	15.4	0	28.6	0.88	0.32	0.085
AIH	4.6	7.1	16.1	7.1	0.64	**0.002**^**c**^	0.64
Cholangitis	2.0	0	45.2	14.3	0.99	**< 0.001**^**d**^	0.0**17**^**e**^
**Median time between**							
Diagnosis* of bowel disorder and diagnosis of cirrhosis	/	12 ± 5	11 ± 4	13 ± 5	/	/	/
**Median at diagnosis of**							
cirrhosis (years)	50 ± 15	46 ± 11	47 ± 12	48 ± 9	/	/	/

Quantitative measures are expressed as median with IQR or relative frequency (%). *ALT* alanine aminotransferase, *ALP* alkaline phosphatase, *AST* aspartate aminotransferase, *BMI* body mass index, *GGT* gamma-glutamyl transferase, *NASH* Non-alcoholic steatohepatitis, *ULN* upper limit of normal (sex-specific). ^a^OR = 0.24[0.08–0.74]; ^b^OR = 8.28[3.82–20.2]; ^c^OR = 4.04[1.48–11.00]; ^d^OR = 37.69[15.69–90.55]; ^e^OR = 7.37[1.43–37.94]. p-values <0.05 are highlighted in bold. *Refers to age at baseline examination. Some of the diagnoses were obtained from previous medical records since they were made prior to the baseline examination.

To further characterize the factors associated with the development of cirrhosis in different intestinal disease entities, we compared cirrhotics with non-cirrhotics. Among the CeD cohort, cirrhotics more frequently displayed NASH (OR = 98.29 [16.42–588.51]; p < 0.001) or AIH (OR = 36.28 [3.96–332.40]; p < 0.001), and they were more frequently obese or had diabetes (Supplementary Table 4). Among participants with UC and cirrhosis, cholangitis (OR = 54.89 [25.75–116.97]; p < 0.001) and AIH (OR = 175.43 [44.57–690.53]; p < 0.001) were clearly overrepresented, while diabetes played a less prominent role (Supplementary Table 5). Among the CD cohort, cholangitis (OR = 41.31 [7.77–219.64]; p < 0.001), NASH (OR = 689.20 [70.37–6723.02]; p < 0.001) and AIH (OR = 26.5 [2.9–242.4]; p < 0.001) were all markedly overrepresented in cirrhotics vs. non-cirrhotics (Supplementary Table 6). In line with the prominent role of NASH, the CD cirrhotics more frequently had diabetes and were obese (Supplementary Table 6). The analysis of the control cohort reflected the well-established factors associated with development of cirrhosis, i.e. higher age, male sex, alcohol consumption obesity, diabetes mellitus as well as presence of liver co-morbidities (Supplementary Table 7).

## Discussion

In our study, we analyzed the hepatobiliary phenotype of the most common inflammatory intestinal diseases using the well-characterized community sample available in the UK Biobank. By this approach, we demonstrated elevated transaminases in CeD compared to controls. This is not surprising, since CeD subjects were shown previously to more frequently display elevated transaminases than the general population even when adhering to a strict gluten-free diet^30^. For example, Castilo et al. reported elevated transaminases to be ~ 1.5 times more common in individuals with CeD even 1.5 years after the start of gluten-free diet compared to matched controls^31^. Notably, the frequency of elevated transaminases in the CeD cohort reported herein is lower than that in the previous studies (i.e. < 10%), which might be due to the facts that (1) we excluded subjects with various liver co-morbidities and (2) the UK biobank is enriched for healthy individuals^29^. On the other hand, our observation that average transaminase levels do not substantially differ between the IBD cohort and healthy controls is novel, since no robust, community-based data exist on this topic. This is unexpected, since several studies demonstrated that abnormal liver tests are common in patients with CD and UC^32^ and several liver diseases are overrepresented in individuals with IBD^28,33^. While these data add the population-based perspective and strengthen the importance of an appropriate clinical work-up in IBD individuals with elevated serum transaminases, they also have several important limitations. Because of that, further studies are needed to define the values in phases of active inflammation vs. remission, the impact of different treatment regimen and many more.

In contrast to serum transaminases, the CeD cohort did not display elevated GGT levels. This is interesting, since CeD was previously shown to increase the risk of liver steatosis^26,34^ and in our study, the CeD cohort more frequently harbored NASH. However, the CeD cohort also had lower alcohol consumption, lower BMI values and relatively low percentage of diabetic subjects, which are all conditions associated with lower GGT values^35,36^. Another unexpected finding were the elevated ALP levels in the CeD cohort compared to controls. In this respect, several studies suggested that increased ALP values are uncommon in CeD and might be related to bone disease rather than cholestatic disorders^30^. Although bone affection might be the key determinant of elevated ALP values in CeD individuals, in our study, the CeD cohort also more frequently suffered cholelithiasis. While the association between gallstones and CeD has not been established previously^26^, CeD subjects were reported to have impaired gallbladder motility, which constitutes a well-established factor predisposing to gallstone formation^37^. Finally, CeD individuals are at a higher risk for primary biliary cirrhosis^26^ and this established association might also be in part responsible for elevated ALP levels.

In patients with CD/UC, we observed elevated GGT and ALP concentrations. A likely explanation for the former finding is that steatosis is overrepresented in both disorders and was shown to correlate with severity of colitis and duration of disease^26,33,38^. This is likely in part due to steroid use. With regard to the elevated ALP levels, a simultaneous occurrence of PSC is particularly important in UC and may be seen in up to 8% of cases, whereas it is less common in CD^39,40^. On the other hand, gallstones are more prevalent in CD individuals, which is well in line with our observations^28^. Notably, we also saw a higher rate of gallstones in UC individuals, however, this finding is controversial^26,28^ and needs to be confirmed by future studies.

Beyond looking at the importance of individual diseases, we assessed the impact of previous intestinal resections. In CD, this event was associated with elevated AST, ALT and ALP levels as well as higher occurrence of gallstones. This is not surprising, since inflammation and/or removal of terminal ileum leads to loss of bile acids that are crucial to prevent cholesterol precipitation^19,21^. In UC, the impact of previous surgery was even more striking and was associated with higher AST, ALT, GGT and ALP concentrations as well as higher occurrence of gallstones and cholangitis, presumably referring to PSC. This is in line with a previous report that demonstrated high frequency of abnormal liver enzymes in individuals with ileal pouch-anal anastomosis^41^. Several reasons might be responsible for this observation. First, surgery is substantially less common in UC than CD and accordingly, it indicates a small subset of patients with a severe, more generalized disease. Moreover, a simultaneous presence of PSC and UC is associated with more severe colitis^28^, that likely accounts for higher surgery rates in individuals suffering from both diseases. Finally, colectomy was shown to alter the biochemical composition of the bile and this mechanism might be responsible for the higher frequency of gallstones^42^.

While the liver enzyme values differed between the analyzed intestinal disorders, all three conditions resulted in a comparably increased occurrence of liver cirrhosis. In the case of CeD, the adjusted OR of ~ 3.6 seen in this study is well in line with previously published data suggesting that CeD subjects display a three times increased hepatic mortality^43^. Two other studies also found at least twice elevated prevalence of liver fibrosis/cirrhosis in CeD individuals compared to age- and sex-matched controls^12,44^. This is likely at least in part due to increased occurrence of chronic hepatitis, non-alcoholic steatohepatitis and autoimmune hepatitis that were found both in our study and previous reports^26,34,44^. Conversely, increased cirrhosis rates in UC are primarily due to the increased prevalence of PSC and to lesser extent to autoimmune hepatitis and steatosis^28^. This may also explain the higher prevalence of HCCs in the CeD but not in the UC cohort^39^. Vice versa, compared to UCs, liver cirrhosis in CDs might be more related to liver steatosis and less to PSC, which may explain the higher occurrence of HCCs. In contrast to our findings, a recent analysis described a similarly increased risk of HCC in CD and UC individuals^45^. Given that the design of UK biobank cohort does not allow a careful analysis of the medical charts of the individual patients and since the diagnosis of HCC and CCA might be difficult to discern, further studies are needed to corroborate our findings. Finally, drug-induced liver injury is known to play a role in both CD and UC^28^, however, cannot be reliably assessed with the data available in the UK Biobank.

Our study has both significant strengths and limitations. Its cross-sectional design precludes an identification of causal relationships and is not well-suited for assessing rapidly progressive disorders such as cholangiocellular carcinoma. In addition, the diagnosis of the studied diseases is based on UK hospital admission codes (ICD10), which may miss some patients, in particular in the case of CeD. However, previous analyses used the same approach and saw a similar performance as case–control studies^46,47^.

A major advantage of the UK Biobank cohort is its community-based setting that closely mimics the general population and minimizes a selection bias seen in single-center studies. Moreover, it allows side-by-side comparison of the different intestinal disorders, which is otherwise difficult to accomplish. Moreover, our study quantifies the previously suggested association between chronic intestinal disorders and the occurrence of end-stage liver disease. This association should promote a more thorough hepatologic monitoring of individuals with these intestinal disorders, especially in situations with recurrently elevated liver enzymes and/or presence of additional risk factors, such as obesity, diabetes or metabolic syndrome.

## Supplementary Information

Supplementary Information.

## Data Availability

The data analyzed in this article are property of UK Biobank and can be obtained through a procedure described at http://www.ukbiobank.ac.uk/using-the-resource/.

## Abbreviations

AIH: Autoimmune hepatitis
ALP: Alkaline phosphatase
ALT: Alanine aminotransferase
AST: Aspartate aminotransferase
BMI: Body mass index
CCA: Cholangiocarcinoma
CD: Crohn’s disease
CeD: Celiac disease
CI: Confidence Interval
DM: Diabetes mellitus
GGT: Gamma glutamyl transferase
HCC: Hepatocellular carcinoma
IQR: Interquartile range
NASH: Nonalcoholic steatohepatitis
OR: Odds ratio
PSC: Primary sclerosing cholangitis
SD: Standard deviation
UC: Ulcerative colitis
UKB: United Kingdom Biobank
ULN: Upper limit of normal
