# Supplementary material for: Hepatobiliary phenotype of individuals with chronic intestinal disorders
Source: Sci Rep. 2021 Oct 7;11:19954. doi: 10.1038/s41598-021-98843-7 (PMC8497585; doi:10.1038/s41598-021-98843-7)
Supplement: Supplementary file 1 — Supplementary Information. [file 41598_2021_98843_MOESM1_ESM.docx]

**Supplementary Material of**

**Hepatobiliary Phenotype of Individuals with Chronic Intestinal Disorders**

**Short title:** Hepatobiliary Changes in Intestinal Disorders

Jessica Voss^1*^, Carolin V. Schneider^1*^, Moritz Kleinjans^1^, Tony Bruns MD^1^, Christian Trautwein MD^1^, Pavel Strnad, MD^1#^

**Population-based UK Biobank participants**

**Exclusion of chronic viral hepatitis, pathological alcohol consumption or diagnosis of more than one IBD/CeD diseases (n=5 771)**

**Ulcerative colitis**

n= 3 684

**Crohn’s disease**

n= 1 738

**Coeliac disease**

n= 2 377

**Analysis of gut-liver axis related parameters**

**Supplementary Figure 1: Overview of the analyzed cohort.**

Population-based study analyzing UK Biobank participants of European ancestry aged 37 to 73 years.

|  | **Controls**  *n=488.941* | **Celiac disease (CeD)**  *n=2 377* | **Ulcerative colitis (UC)**  *n=3 684* | **Crohn (CD)**  *n=1 738* | **p-value**  **CeD vs. Controls** | **p-value**  **UC vs. Controls** | **p-value**  **CD vs. Controls** |
| --- | --- | --- | --- | --- | --- | --- | --- |
| **Liver status** |  |  |  |  | *Multivariable* | *Multivariable* | *Multivariable* |
| ALT (% of ULN) | 56.3±32.6 | 61.6±40.7 | 56.3±35.4 | 56.1±32.7 | **1.1×10^-26^** | .37 | .44 |
| ALT ≥ULN (%) | 6.8 | 8.7 | 7.1 | 7.8 | **3.9×10-10^a^** | .28 | **.019^b^** |
| AST (% of ULN) | 63.6±25.7 | 72.3±35.3 | 64.3± 31.3 | 64.7±24.1 | **2.0×10-47** | **.0004** | .070 |
| AST ≥ULN (%) | 4.6 | 9.8 | 5.7 | 6.3 | **3.6×10-28^c^** | **.001^d^** | **.001^e^** |
| GGT (% of ULN) | 75.2±80.5 | 73.9±94.1 | 88.7±125.8 | 82.9± 101.4 | .072 | **1.4×10-23** | **7.5×10-8** |
| GGT ≥ULN (%) | 17.1 | 15.4 | 20.3 | 21.1 | .47 | **4.3×10-7^f^** | **9.3×10-10^g^** |
| ALP (% of ULN) | 72.7±24.6 | 79.0±32.0 | 77.3±41.0 | 80.8±29.5 | **3.3×10-18** | **1.1×10-40** | **1.9×10-41** |
| ALP ≥ULN (%) | 11.6 | 17.6 | 15.1 | 19.8 | **6.0×10-9^h^** | **1.5×10-15^i^** | **9.9×10-22^j^** |
| Bilirubin (mg/dl) | 0.53±0.26 | 0.48±0.25 | 0.54±0.27 | 0.51±0.26 | **4.9×10-15** | .36 | **.003** |
| Bilirubin≥ULN (%) | 2.9 | 2.5 | 3.4 | 2.9 | .31 | .23 | .95 |
| Albumin (g/l) | 45.2±2.6 | 44.6±2.8 | 44.7±2.8 | 43.9± 3.3 | **1.1×10-21** | **7.5×10-29** | **1.7×10-87** |

**Supplementary Table 1: Liver status in individuals with celiac disease, Crohn’s disease or ulcerative colitis compared to controls.**

Quantitative measures are expressed as mean with standard deviation or relative frequency (%). Multivariable analyses were adjusted for age, sex, BMI, presence of diabetes mellitus, and mean alcohol consumption. Color-coding was used to mark p-values 0.001-0.05 in yellow and p-value <0.001 in orange. Abbreviations: ALT, alanine aminotransferase; ALP, alkaline phosphatase; AST, aspartate aminotransferase; BMI, body mass index; GGT, gamma-glutamyl transferase; ULN, upper limit of normal (sex-specific). ^a^aOR=1.62[1.39-1.89]; ^b^aOR=1.24[1.04-1.50]; ^c^aOR=2.24[1.94-2.58]; ^d^aOR=1.29[1.11-1.50]; ^e^aOR=1.43[1.17-1.75]; ^f^aOR=1.25[1.15-1.36]; ^g^aOR=1.47[1.30-1.66]; ^h^aOR=1.41[1.26-1.59]; ^i^aOR=1.50[1.36-1.66]; ^j^aOR=1.90[1.67-2.17].

|  | **Controls**  *n=488 941* | **Coeliac disease (CeD)**  *n=2 377* | **Ulcerative colitis (UC)**  *n=3 684* | **Crohn (CD)**  *n=1 738* | **p-value**  **CeD vs. Controls** | **p-value**  **UC vs. Controls** | **p-value**  **CD vs. Controls** |
| --- | --- | --- | --- | --- | --- | --- | --- |
| **Liver status** |  |  |  |  | *Multivariable* | *Multivariable* | *Multivariable* |
| ALT ≥ULN (%) | 6.8 | 8.7 | 7.1 | 7.8 | **3.9×10-10^a^** | .28 | **.019^b^** |
| ALT ≥2x ULN (%) | 0.61 | 0.72 | 0.75 | 0.99 | .39 | .17 | **.036^c^** |
| AST ≥ULN (%) | 4.6 | 9.8 | 5.7 | 6.3 | **3.6×10-28^d^** | **.001^e^** | **.001^f^** |
| AST ≥2x ULN (%) | 0.34 | 0.54 | 0.61 | 0.31 | .065 | **.005^g^** | .92 |
| GGT ≥ULN (%) | 17.1 | 15.4 | 20.3 | 21.1 | .47 | **4.3×10-7^h^** | **9.3×10-10^i^** |
| GGT ≥2x ULN (%) | 4.2 | 4.1 | 6.0 | 5.6 | .31 | **6.3×10-8^j^** | **.0002^k^** |
| GGT ≥5x ULN (%) | 0.55 | 0.90 | 1.59 | 0.99 | **.005^l^** | **1.6×10-15^m^** | **.006^n^** |
| ALP ≥ULN (%) | 11.6 | 17.6 | 15.1 | 19.8 | **6.0×10-9^o^** | **1.5×10-12^p^** | **9.9×10-22^q^** |
| ALP ≥2x ULN (%) | 0.17 | 0.81 | 0.72 | 0.68 | **2.3×10-9^r^** | **4.9×10-12^s^** | **.00001^t^** |

**Supplementary Table 2: Elevated liver enzymes in individuals with Coeliac disease, Crohn’s disease or ulcerative colitis compared to controls.**

Quantitative measures are expressed as mean with standard deviation or relative frequency (%). Multivariable analyses were adjusted for age, sex, BMI, presence of diabetes mellitus, and mean alcohol consumption. Abbreviations: ALT, alanine aminotransferase; ALP, alkaline phosphatase; AST, aspartate aminotransferase. ^a^aOR=1.62[1.39-1.89]; ^b^aOR=1.24[1.04-1.50]; ^c^aOR=1.70[1.03-2.79]; ^d^aOR=2.24[1.94-2.58]; ^e^aOR=1.29[1.11-1.50]; ^f^aOR=1.43[1.17-1.75]; ^g^aOR=1.86[1.21-2.87]; ^i^aOR=1.25[1.15-1.36]; ^j^aOR=1.47[1.30-1.66]; ^k^aOR=1.49[1.29-1.72]; ^l^aOR=1.50[1.21-1.86]; ^m^aOR=1.89[1.21-2.94]; ^n^aOR=3.01[2.29-3.94]; ^o^aOR=1.41[1.26-1.59]; ^p^aOR=1.50[1.36-1.66]; ^q^aOR=1.90[1.67-2.17]; ^r^aOR=4.20[2.62-6.72]; ^s^aOR=4.22[2.81-6.36]; ^t^aOR=3.81[2.09-6.36]; ^´^

|  | **Only small bowel Crohn (sCD)**  *n=415* | **Only Large bowel Crohn (lCD)**  *n=583* | **p-value**  **CD vs. Controls** |
| --- | --- | --- | --- |
|  | |  | *Multivariable* |
| **Liver status** |  |  |  |
| ALT (% of ULN) | 56.5±33.38 | 57.11±37.56 | .79 |
| ALT ≥ULN (%) | 9 | 8 | .54 |
| AST (% of ULN) | 64.67±27.18 | 67.41±31.45 | .30 |
| AST ≥ULN (%) | 7 | 9 | .58 |
| GGT (% of ULN) | 81.39±77.94 | 93.77±146.54 | .22 |
| GGT ≥ULN (%) | 22 | 22 | .85 |
| ALP (% of ULN) | 81.84±27.63 | 81.4±39.98 | .66 |
| ALP ≥ULN (%) | 22 | 19 | .25 |
| **ICD10 coded hepatobiliary diseases** |  |  |  |
| Cirrhosis (%) | 0.84 | 0.81 | .93 |
| Chronic Hepatitis (%) | 0.24 | 0.51 | .56 |
| AIH (%) | 0.24 | 0.86 | .24 |
| NASH (%) | 0.48 | 0.17 | .35 |
| Cholelithiasis (%) | 15.2 | 8.6 | **.001^a^** |
| Cholecystitis (%) | 2.1 | 2.4 | .55 |
| Cholangitis (%) | 1.45 | 1.20 | .85 |
| HCC (%) | 0.00 | 0.51 | .99 |
| CCA (%) | 0.24 | 0.17 | .92 |

**Supplementary Table 3: Liver phenotype in individuals with small and large bowel Crohn’s disease.**

Quantitative measures are expressed as mean with standard deviation or relative frequency (%). Multivariable analyses were adjusted for age, sex, BMI, presence of diabetes mellitus, and mean alcohol consumption. Abbreviations: BMI, body mass index; CCA; cholangiocarcinoma: NASH, Non-alcoholic steatohepatitis.Small vs. large bowel crohn ^a^aOR=1.94[1.30-2.90].

|  | **Coeliac disease**  *without cirrhosis*  *n=2 363* | **Coeliac disease**  *with cirrhosis*  *n=14* | **p-value** |
| --- | --- | --- | --- |
| **Characteristics** |  |  |  |
| Age (years) | 57.9±7.8 | 59.7±4.8 | .19 |
| Women (%) | 65 | 43 | .076 |
| BMI (kg/m^2^) | 26.1±4.8 | 28.1±5.1 | .17 |
| Alcohol (g/d) | 6.5±8.4 | 9.7±17.9 | .52 |
|  |  |  |  |
| **Risk factors** |  |  |  |
| BMI>30 kg/m^2 a^ | 21 | 50 | **.008** |
| Diabetes mellitus (%)^b^ | 5.1 | 21.4 | **.006** |
| Type 1 | 2.6 | 7.1 | .29 |
| Type 2^c^ | 2.5 | 14.3 | **.006** |
| NASH^d^ | 0.17 | 14.3 | **9.1*10-26** |
| Cholangitis | 0.30 | 0 | .83 |
| AIH^e^ | 0.21 | 7.1 | **2.6*10-7** |
| HCC^f^ | 0.13 | 7.1 | **1.7*10-10** |

**Supplementary Table 4: Comparison of cirrhotic and non-cirrhotic individuals with Coeliac disease.**

Quantitative measures are expressed as mean with standard deviation or relative frequency (%). Abbreviations: AIH, Autoimmunehepatitis; NASH, Non-alcoholic steatohepatitis.
^a^OR=3.75[1.31-10.73]; ^b^OR=5.12[1.41-18.60]; ^c^OR=6.40[1.40-29.21]; ^d^OR=98.29[16.42-588.51]; ^e^OR=36.28[3.96-332.40]; ^f^OR=60.51[5.90-620.64].

|  | **Ulcerative colitis**  *without cirrhosis*  *n=3 653* | **Ulcerative colitis**  *with cirrhosis*  *n=31* | **p-value** |
| --- | --- | --- | --- |
| **Characteristics** |  |  |  |
| Age (years) | 57.6±7.9 | 60.4±6.5 | **.023** |
| Women (%) | 47 | 32 | .085 |
| BMI (kg/m^2^) | 27.5±4.7 | 28.2±4.1 | .42 |
| Alcohol (g/d) | 8.4±10.1 | 8.4±13.9 | .99 |
|  |  |  |  |
| **Risk factors** |  |  |  |
| BMI>30 kg/m^2^ | 32.6 | 35.5 | .73 |
| Diabetes mellitus (%) | 7.5 | 19.4 | .068 |
| Type 1^a^ | 2.0 | 9.7 | **.003** |
| Type 2 | 5.5 | 9.7 | .30 |
| NASH | 0.2 | 0 | .82 |
| Cholangitis^b^ | 1.5 | 45.2 | **5.8*10-13** |
| AIH^c^ | 0.1 | 16.1 | **2.3*10-72** |
| HCC^d^ | 0.08 | 3.23 | **8.8*10-36** |

**Supplementary Table 5: Comparison of cirrhotic and non-cirrhotic individuals with ulcerative colitis.**

Quantitative measures are expressed as mean with standard deviation or relative frequency (%). Abbreviations: AIH, Autoimmunehepatitis; NASH, Non-alcoholic steatohepatitis. ^a^OR=5.18[1.54-17.43]; ^b^OR=54.89[25.75-116.97]; ^c^OR=175.43[44.57-690.53]; ^d^OR=251.86[22.22-2855.46].

|  | **Crohn’ disease**  *without cirrhosis*  *n=1 724* | **Crohn’s disease**  *with*  *cirrhosis n=14* | **p-value** |
| --- | --- | --- | --- |
| **Characteristics** |  |  |  |
| Age (years) | 56.6±8.1 | 58.1±6.8 | .42 |
| Women (%) | 57 | 64 | .59 |
| BMI (kg/m^2^) | 27.0 ± 4.9 | 29.4±4.1 | .059 |
| Alcohol (g/d) | 6.7±9.0 | 4.4±8.8 | .34 |
|  |  |  |  |
| **Risk factors** |  |  |  |
| BMI>30 kg/m^2 a^ | 28.3 | 62 | **.008** |
| Diabetes mellitus (%)^b^ | 7.0 | 35.7 | **.00002** |
| Type 1^c^ | 1.7 | 21.4 | **4.4*10-8** |
| Type 2 | 5.3 | 14.3 | .14 |
| NASH^d^ | 0.06 | 28.6 | **6.2*10-100** |
| Cholangitis^e^ | 0.4 | 14.3 | **2.2*10-72** |
| AIH^f^ | 0.3 | 7.1 | **.00001** |
| HCC^g^ | 0.12 | 14.3 | **3.1*10-28** |

**Supplementary Table 6: Comparison of cirrhotic and non-cirrhotic individuals with Crohn’s disease.**

Quantitative measures are expressed as mean with standard deviation or relative frequency (%). Abbreviations: AIH, Autoimmunehepatitis; NASH, Non-alcoholic steatohepatitis. ^a^OR=4.05[1.32-12.43]; ^b^OR=7.74[2.55-23.47]; ^c^OR=15.9[4.22-60.17]; ^d^OR=689.20[70.37-6723.02]; ^e^OR=41.31[7.77-219.64]; ^f^OR=26.45[2.89-242.35]; ^g^OR=143.50[18.65-1104.20].

|  | **Controls**  *without cirrhosis n=487 929* | **Controls**  *with*  *cirrhosis*  *n=1 012* | **p-value** |
| --- | --- | --- | --- |
| **Characteristics** |  |  |  |
| Age (years) | 56.5±8.1 | 59.1±6.9 | **1.2*10-29** |
| Women (%)^a^ | 55 | 31 | **7.2*10-53** |
| BMI (kg/m^2^) | 27.4±4.8 | 30.3±6.1 | **1.2*10-43** |
| Alcohol (g/d) | 8.8±10.1 | 10.8±14.4 | **.000007** |
|  |  |  |  |
| **Risk factors** |  |  |  |
| BMI>30 kg/m^2 b^ | 31.0 | 53.3 | **5.6*10-52** |
| Diabetes mellitus (%)^c^ | 5.3 | 28 | **4.5*10-224** |
| Type 1^d^ | 0.8 | 7.3 | **1.3*10-121** |
| Type 2^e^ | 4.5 | 21.5 | **2.4*10-150** |
| NASH^f^ | 0.04 | 12.9 | **3.0*10-300** |
| Cholangitis^g^ | 0.14 | 2.0 | **1.1*10-53** |
| AIH^h^ | 0.03 | 4.6 | **3.0*10-300** |
| HCC^i^ | 0.02 | 7.81 | **3.0*10-300** |

**Supplementary Table 7: Comparison of cirrhotic and non-cirrhotic control individuals.**

Quantitative measures are expressed as mean with standard deviation or relative frequency (%).^a^OR=2.73[2.39-3.12]; ^b^OR=2.54[2.24-2.88]; ^c^OR=7.74[2.55-23.47]; ^d^OR=10.08[7.94-12.79]; ^e^OR=5.86[5.04-6.81];^f^OR=373.83[296.64-471.11];^g^OR=14.43[9.21-22.60]; ^h^OR=147.94[105.87-206.74]; ^i^OR=372.12[276.90-500.08].
